# Sequential flaps reconstruction in head and neck cancer: A systematic review

**DOI:** 10.1016/j.bjorl.2025.101693

**Published:** 2025-07-29

**Authors:** Yu Xiong, Zepeng Xu, Mailudan Ainiwaer, Zheng Jiang, Fei Chen

**Affiliations:** a Sichuan University, West China Hospital, Department of Otolaryngology, Head and Neck Surgery, Chengdu, China; b Sichuan University, West China Hospital, Head and Neck Surgical Center, Chengdu, China; c West China Clinical Medical College of Sichuan University, Chengdu, China

**Keywords:** Sequential flaps, Head and neck cancer, Reconstruction

## Abstract

**Objectives:**

For patients with Head and Neck Cancer (HNC), flap reconstruction is one of the treatment modalities for large head and neck defects. While most patients achieve satisfactory outcomes with a single flap, some may require a second or additional flaps due to various reasons. The aim of our study was to systematically review the relevant articles and investigate the application of sequential flaps in reconstruction of patients with HNC.

**Methods:**

Two authors independently screened articles from PubMed, EBSCO, and Web of Science. Studies were included if they reported at least one of the following outcomes: duration of operation or hospitalization, flap-related postoperative complications, flap success rate, and follow-up outcomes.

**Results:**

A total of 17 articles were included, comprising 832 patients and 1037 flaps (excluding the number of flaps used in initial reconstructions). The overall flap failure rate was 5.4%. Among the 314 flap-related postoperative complications, the most common were revision (5.8%), complete flap necrosis (5.2%), and arterial or venous thrombosis (3.3%).

**Conclusion:**

Sequential flaps are an optional and effective reconstructive modality for HNC patients. However, prospective studies are needed to explore prognostic-related risk factors and better determine the suitability of sequential flaps for individual patients. Additionally, since reconstruction impacts both aesthetics and function, it is crucial to emphasize during preoperative discussions that postoperative outcomes may not fully align with patient expectations.

## Highlights

• Sequential flaps can be used as an effective method for head and neck reconstruction.
• The survival rate of sequential flaps was 94.6%.
• The anterolateral thigh flap is the mostly used sequential flap type.

## Introduction

Head and Neck Cancer (HNC) are epithelial malignant tumors that occur in the upper aero digestive tract, including the nasal cavity, sinuses, oral cavity, pharynx, and larynx. The primary risk factors for HNC are long-term heavy alcohol consumption and cigarette smoking. In addition, Human Papilloma Virus (HPV) is also a well-established risk factor for HNC. Recent reports demonstrate that the number of patients with tobacco related HNC is slowly decreasing globally due to the decreasing use in tobacco.^1^^,^^2^ However, HNC is still the sixth most common tumor in the world, with over 870,000 new cases and 440,000 deaths in 2020.^3^

In recent years, the treatment options for HNC are no longer limited to surgery, traditional radiotherapy and chemotherapy, new treatment methods such as Proton Beam Therapy (PBT), personalized radiotherapy, targeted immunotherapy, and de-intensified therapy provide more possibilities for the treatment of HNC.^4^, ^5^, ^6^, ^7^ Despite this, surgical resection remains the most vital treatment option. Soft-tissue (e.g., mucosal, skin, etc.) defects occurring after resection often require concurrent reconstruction surgery, and for larger defects, microsurgical tissue transplantation is a reliable method to repair. The objective of reconstruction is to restore tissue loss and repair the soft-tissue defects with a view to enabling wound healing and the recovery of a relatively normal appearance and function to ensure the quality of life for patients.

However, due to the potential risks of for flap necrosis, tumor recurrence, or poor recovery of associated function following the initial flap reconstruction, some studies have proposed and evaluated the use of sequential flaps for head and neck reconstruction. For surgeons, determining when and how to select sequential flaps and anastomotic vessels is critical to the success of the reconstruction. For patients, they are more concerned with the restoration of a relatively normal appearance and associated function. Therefore, we aim to conduct a systematic review of the articles on sequential flaps in reconstructive surgery for HNC patients, to summarize the indication of re-operation, the selection of flaps and vessels, outcomes and complications, to assess whether sequential flaps can be considered a safe and reliable option when previous flaps are unavailable for various reasons. To achieve this, we will undertake a systematic review of the relevant articles.

## Methods

This review was conducted in the recommendations of the PRISMA (Preferred Reporting Items for Systematic Evaluation and Meta-Analysis) 2009 Guidelines and the PRISMA 2020 Updated Guideline.^8^^,^^9^

### Information sources and search strategy

A comprehensive search of relevant electronic articles was conducted independently by two authors. Databases searched included PubMed (www.pubmed.org), Web of Science (www.webofscience.com), EBSCO (www.ebsco.com), running by the following keywords: “free flap AND head and neck AND reconstruction AND (second OR sequential)” through to September 2023.

### Eligibility criteria selection process

**Fig. 1:**
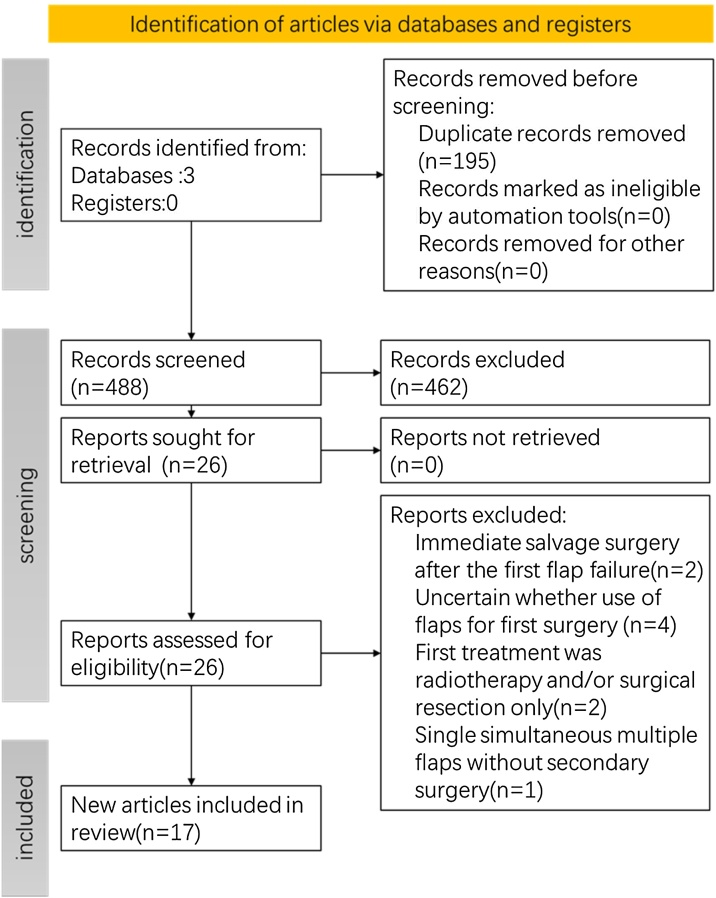
Flow diagram of articles screening process.

All relevant published articles were systematically summarized and screened following the inclusion and exclusion criteria shown in Table 1. The initial search returned 683 results. After removing duplicate articles using EndNote software, two authors conducted an independent initial screening based on predetermined inclusion criteria through titles and abstracts to ensure a comprehensive range of relevant literature was considered. Next, full-text reading was performed, and the articles included in this systematic review was finally determined by reading the full text of the initially screened articles, performing secondary screening based on the exclusion criteria, and then manually searching for references in these selected articles. The process of articles screening is shown in Fig. 1.

**Table 1:** Inclusion and exclusion criteria.

Inclusion criteria	Exclusion criteria
Patients with head and neck cancer undergoing sequential flaps reconstruction	First treatment was radiotherapy/chemotherapy and/or surgical resection only
All flaps were used for head and neck reconstruction	Single simultaneous multiple flaps without secondary surgery
Include the type of sequential flaps and vessels	Uncertain whether the use of flaps for the first surgery
At least one of the following outcomes: operation time or hospitalization, flaps related postoperative complications, success rate of flaps and follow-up outcomes	Immediate salvage surgery after the first flap failure
Non-Review Articles	
English article

### Data collection process and quality assessment

The required data were extracted from the full text of the article, and the collected data included: mean age of patients, number of patients and flaps, indication of sequential reconstruction, comorbidities (e.g., diabetes), types of flaps used for each reconstruction, vessels used for anastomosis in each reconstruction, postoperative complications, and patients outcomes including mean follow-up time, number of survivors at the last follow-up, 5-year survival rate, operation time and hospitalization duration.

The level of evidence was assessed by the Oxford Center for Evidence-Based Medicine (OCEBM) 2011 Levels of Evidence,^10^ with levels ranging from Ⅰ to V, and level Ⅰ is the highest level. The risk of bias was assessed by the Methodological Index for Non- Randomized Studies (MINORS) criteria.^11^ The MINORS criteria items are scored 0 (not reported), 1 (reported but inadequate) and 2 (reported and adequate). The global ideal score for non-comparative studies is 16, and for comparative studies is 24. Non-comparative studies were considered to be at high risk of bias if the total score less than 10 (the comparative studies less than 18), modern risk for bias if the total score between 10 and 14 (the comparative studies between 18 and 22), low risk for bias if the total score was 15 or 16 (the comparative studies 23 or 24).

## Results

A total of 683 results were obtained, of which 195 were identified as duplicates and subsequently excluded. Based on titles and abstracts, 26 articles were selected for full-text review, and then further screening based on exclusion criteria resulted in 17 articles^12^, ^13^, ^14^, ^15^, ^16^, ^17^, ^18^, ^19^, ^20^, ^21^, ^22^, ^23^, ^24^, ^25^, ^26^, ^27^, ^28^ (Fig. 1). The 17 articles included in this review were published between July 1995 and November 2021, and from 7 different countries. All included articles were retrospective case-series or case-control studies, with a level of evidence rated as Ⅳ according to OCEBM 2011. Among these, only 2 articles^20^^,^^21^ were comparative studies, while the remaining 15 were non-comparative studies. Among the 15 non-comparative studies, 14 were assessed as having a moderate risk of bias, and 1 was assessed as having a high risk of bias. The two comparative studies were both assessed as having a moderate risk of bias (Table 2).

**Table 2:** Methodological quality of included articles by MINORS criteria.

Articles	MINORS criteria												Scores
	Un-comparative study^a^								Addition^b^				
	1	2	3	4	5	6	7	8	1	2	3	4	
Moratin et al.^12^	2	2	2	2	0	1	2	1	–	–	–	–	12
Lai et al.^13^	2	2	2	2	1	2	2	1	–	–	–	–	14
Jung et al.^14^	2	2	2	2	0	2	2	0	–	–	–	–	12
Vamadeva et al.^15^	2	2	2	0	0	1	2	0	–	–	–	–	9
Chung et al.^16^	2	2	2	2	0	2	2	1	–	–	–	–	13
Chiu et al.^17^	2	2	2	2	1	2	2	1	–	–	–	–	14
Mochizuki et al.^18^	2	2	2	2	0	2	2	1	–	–	–	–	13
Hanasono et al.^19^	2	2	2	2	0	2	2	1	–	–	–	–	13
Ross et al.^20^^c^	2	2	2	2	1	1	2	1	2	2	2	2	21
Alam et al.^21^^c^	2	2	2	2	1	2	2	0	2	2	2	0	19
Mccarn et al.^22^	2	2	2	2	1	2	0	0	–	–	–	–	11
Varvares et al.^23^	2	2	2	2	0	2	2	0	–	–	–	–	12
Knoetgen et al.^24^	2	2	2	2	1	2	0	0	–	–	–	–	11
Nakayama et al.^27^	2	2	2	2	0	2	2	0	–	–	–	–	12
Demirkan et al.^25^	2	2	2	2	0	2	2	0	–	–	–	–	12
Demirkan et al.^28^	2	2	2	2	0	2	2	0	–	–	–	–	12
Pickford et al.^26^	2	2	2	2	0	2	2	0	–	–	–	–	12

a The criteria of un-comparative study as follows:1 - Clearly stated aim, 2 - Inclusion of consecutive patients, 3 - Endpoints appropriate to the aim of the study, 4 - Unbiased assessment of the study endpoint, 5 - Unbiased assessment of the study endpoint, 6 - Follow-up period appropriate to the aim of the study, 7 - Loss to follow up less than 5%, 8 - Prospective calculation of the study size.

b The additional criteria of comparative study as follows: 1 - An adequate control group, 2 - Contemporary groups, 3 - Baseline equivalence of groups. 4 - Adequate statistical analyses.

c This article is comparative study, the score includes the additional criteria.

This review encompassed 832 patients, of whom 574 (69%) were male and 258 (31%) were female. The mean age at the time of the second reconstruction was reported in 9 articles,^12^^,^^14^^,^^16^^,^^17^^,^^20^^,^^22^^,^^23^^,^^27^^,^^28^ involving 579 patients, and was 59.4 years. The indication for the first flap reconstruction included HNC, osteoradionecrosis, defects caused by infection and gunshot wound. HNC was the most common indication, accounting for 633 (98.4%) of 643 patients with a detailed report of initial indication. The predominant pathological manifestation was Squamous Cell Carcinoma (SCC). The recurrence of primary cancer was the most common indication of sequential reconstructions, and all patients reported in 6 articles^16^^,^^17^^,^^22^^,^^24^^,^^25^^,^^28^ had sequential reconstructive surgery because of cancer recurrence. The mean interval times between reconstructions were reported in 13 articles,^12^^,^^14^^,^^16^, ^17^, ^18^, ^19^^,^^22^, ^23^, ^24^, ^25^, ^26^, ^27^, ^28^ with the shortest of 12-months and the longest of 49.2-months (Table 3).

**Table 3:** Overview of basic characteristics included articles.

Articles	Study type	Continent (country)	Nº of patients	Sex (M/F)	Mean age^c^ (years)	Nº of 2^nd^ flap	Nº of sequential flaps^a^	First flap indication (nº)	Sequential flaps indication (nº.)	Mean interval time (Mon)
Moratin et al.^12^	Retrospective case series	Europe (Germany)	189	120/69	60	186^b^	220	HNSCC (166)	Recurrence (79)	13.5
								Others (23)	Secondary reconstruction (72)	
									Loss of prior flap (37)	
									Complication (32)	
Lai et al.^13^	Retrospective case series	Asia (China)	36	33/3	N/A	36	87	OSCC (36)	N/A	NA
Jung et al.^14^	Retrospective case series	Asia (Korea)	25	13/12	64.1	31	31	OSCC (25)	Recurrence (25)	34.1
Vamadeva et al.^15^	Retrospective case series	Europe (UK)	17	9/8	N/A	17	39	HNSCC (12)	^#^Recurrence (6)	N/A
								Sarcoma (2)	Infection (6)	
								Infection (2)	Fistula (3)	
								Neuroblastoma (1)	Flap loss (2)	
									Exposed bone/plate (2)	
									Non-union of the mandible (1)	
Chung et al.^16^	Retrospective case series	Asia (Korea)	58	43/15	59	58	58	HNSCC (43)	Recurrence (58)	49.2
								Adenocarcinoma (8)		
								Others (7)		
Chiu et al.^17^	Retrospective case series	Asia (China)	40	38/2	54.6	50	50	Oral cancer (40)	Recurrence (40)	23
Mochizuki et al.^18^	Retrospective case series	Asia (Japan)	26	14/12	N/A	26	57	Oral cancer (25)	Recurrence (12)	12
								Odontogenic tumor (1)	Necrosis (6)	
									Plate fracture or exposure (4)	
									Fistula (2)	
									Modification (1)	
									Secondary cancer (1)	
Hanasono et al.^19^	Retrospective case series	North America (USA)	117	92/25	N/A	130	152	N/A	Recurrent/new (53)	27.8
									Complication (41)	
									Osteoradionecrosis (24)	
									Modification (17)	
									Bony reconstruction for dental implants (3)	
Ross et al.^20^^c^	Retrospective case-control	North America (Canada)	123	74/49	58	123	123	HNSCC (100)	Recurrence (43)	N/A
								Sarcoma (10)	Flap failure (30)	
								Others (13)	Second cancer (23)	
									Complication (27)	
Alam et al.^21^^c^	Retrospective case-control	North America (USA)	33	26/7	N/A	34	34	HNC (25)	Recurrence (19)	N/A
								Osteoradionecrosis (6)	Flap failure (14)	
								Ameloblastoma (1)		
								Gunshot wound (1)		
Mccarn et al.^22^	Retrospective case series	North America (USA)	65	36/29	64.7	66	66	HNSCC (54)	Recurrence (34)	21
								Other tumor (11)	Defect (14)	
									Osteoradionecrosis (8)	
									Metachronous tumor (8)	
									Flap failure (2)	
									Stricture (2)	
Varvares et al.^23^	Retrospective case series	North America (USA)	36	23/13	59.6	36	40	HNC (35)	Recurrence or second tumor (18)	30
								Gunshot wound (1)	Flap failure (6)	
									Defect or modification (10)	
									plate fracture (2)	
									Osteoradionecrosis (3)	
									pharyngoesophageal stenosis (1)	
Knoetgen et al.^24^	Retrospective case series	North America (USA)	12	5/7	N/A	13	13	HNSCC (7)	Recurrence (12)	23
								Others (5)		
								(All tumor)		
Nakayama et al.^27^	Retrospective case series	Asia (Japan)	8	5/3	62	8	8	HNSCC (7)	Recurrence (1)	21
								Osteosarcoma (1)	Second cancer (2)	
									Facial deformity (2)	
									Osteomyelitis (2)	
									Plate exposure (1)	
Demirkan et al.^25^	Retrospective case series	Asia (China)	6	6/0	N/A	6	16	HNSCC (6)	Recurrence(12)	23.8
Demirkan et al.^28^	Retrospective case series	Asia (China)	35	34/1	54	37	37	HNSCC (33)	Recurrence (35)	20
								Adenocarcinoma (1)		
								Verrucous carcinoma (1)		
Pickford et al.^26^	Retrospective case series	Europe (UK)	6	3/3	N/A	6	6	SCC (6)	Recurrence (2)	40
									Second tumor (4)	

HNSCC, Head and Neck Squamous Cell Carcinoma; OSCC, Oral Squamous Cell Carcinoma; CAD, Coronary Artery Disease; CVA, Cerebrovascular Accident; TIA, Transient Ischemic Attack; COPD, Chronic Obstructive Pulmonary Disease; PVD, Peripheral Vascular Disease.

a The number of flaps does not include the number of flaps of the first reconstruction; the number is higher than the number of patients because some patients underwent more reconstructions or multiple flaps were used in a single reconstruction.

b Fewer than the number of patients was that two previous flap reconstructions in the patients included in the study had not been performed at the hospital.

c Age at second reconstruction.

Excluding that initial reconstruction flaps, a total of 1037 flaps were performed in the included patients, of which 981 survived, resulting in sequential flaps survival rate of 94.6%, and a 100% success rate for sequential flaps was reported in 7 articles.^15^^,^^16^^,^^18^^,^^21^^,^^25^, ^26^, ^27^ Among the 1001 flaps for which the type of flap were reported, the most common used flap type was the anterolateral thigh flap (25.9%), followed by the radial forearm flap (22.8%). 14 articles^12^, ^13^, ^14^, ^15^, ^16^, ^17^, ^18^, ^19^^,^^21^^,^^22^^,^^24^, ^25^, ^26^, ^27^ reported details of the arteries used for anastomosis, with the superior thyroid artery being the most frequently used. 12 articles^12^, ^13^, ^14^, ^15^, ^16^^,^^18^^,^^19^^,^^21^^,^^24^, ^25^, ^26^, ^27^ reported veins, with the internal jugular vein (including its branches) being the most common. A more detailed description is provided in Table 4.

**Table 4:** Flaps and vessels reported by articles.

Articles	Type of flap			Success rate (first/sequential) (%)	Type of vessels used for anastomosis					
	First	Sequential;			Arteries			Veins		
		Second	Third or more		First	Sequential		First	Sequential	
						Second	Third or more		Second	Third or more
Moratin et al.^12^	N/A	ALT (81) RFF (34) DCIA (33) Fibula (24) Scapula (9) Composite (5)	ALT (9) RFF (6) DCIA (3) Fibula (10) Scapula (4) Composite (2)	89.9%/ 89.1%	N/A	STA (105) Fac. (27) Lin. (28) ECA (10) TCA (11) APA (4) STempA (1)	STA (10) Fac. (4) Lin. (7) ECA (6) TCA (6) APA (1)	N/A	IJV (141) EJV (27) Fac. (13) Retromandibular (1) Subclavian (1) TCV (2) Brachiocephalic (1)	IJV (22) EJV (7) Fac. (3) TCV (2)
Lai et al.^13^	N/A	N/A	ALT (19) RFF (11) MSAP (11) PTF (4) Fibula (4) Lateral arm (1) DIEP (1)	N/ 94.3%	STA (23) Fac. (10) STempA (3)	STA (12) Fac. (10) STempA (12) Previous pedicle (2)	STA (12) Fac. (7) STempA (14) Previous pedicle (14) TCA (4)	IJV (10) EJV (25) Fac. (10) STV (10) STempV (4)	IJV (8) EJV (13) Fac. (10) STV (11) STempV (14) Previous pedicle (1)	IJV (3) EJV (29) Fac. (3) STV (6) STempV (14) Previous pedicle (13)
Jung et al.^14^	RFF (14) Fibular (7) LD (2) RA (1) ALT (1)	RFF (11) Fibular (6) LD (11) Peroneal artery perforator (1) Dorsalis pedis artery (2)	N/A	97.3%/ 94.6%	STA (12) Lin. (3) Fac. (4) TCA (1) NA (5)	STA (8) Lin. (7) Fac. (13) TCA (2)	N/A	IJV (21) Ant.JV (3) Fac. (1) STV (3) Retromandibular (1) Sug.laryngeal (1)	IJV (19) Fac. (5) STV (4) EJV (5) Retromandibular (1) TCV (2) Thyrolinguofacial trunk (1)	N/A
Vamadeva et al.^15^	RFF (7) ALT (5) Fibular (2) Jejunum (2) DCIA (1)	RFF (6) ALT (7) Fibular (3) Lateral arm (1)	RFF (1) ALT (1) Fibular (3)	100%/ 100%	Fac. (7) STA (2) STempA (1) Occipital (1)	Fac. (7) STA (2) Occipital (2)	N/A	IJV (5) Fac. (5) SJV (1) STempV (1)	IJV (2) Fac. (8) STV (1) EJV (1)	N/A
aChung et al.^16^]	N/A	RFF (10) ALT (11) RA (6) Vastus lateralis (3) LD (2) Fibular (2)	N/A	N/A/ 100%	N/A	STA (26) Lin. (3) Fac. (4) TCA (1)	N/A	N/A	IJV (19) Fac. (4) STV (6) EJV (4) TCV (1)	N/A
Chiu et al.^17^	bALT (27) RFF (8) medial sural flap (4) Fibular (2) RA (1)	ALT (31) RFF (10) Fibular (7) RA (1) Vactus lateralis (1)		92.9%/ 92%	STA (39) Fac. (2)	STA (35) TCA (3) Fac. (5) Lin. (2) Thoracoacromial (1) ECA (2) STempA (2)		N/A		
aMochizuki et al.^18^]	Scapular (9) Forearm (9) Fibular (4) RA (2) LD (1) ALT (1)	Scapular (8) Forearm (6) RA (6) Composite (1)	Scapular (2) Rectus abdominis (2) ALT (1)	N/A/ 100%	N/A	STA (9) TCA (5) Fac. (6) Unknown (1)	STA (1) Fac. (4)	N/A	IJV (1) Fac. (8) STV (3) EJV (6) TCV (1) EJV + Fac. (2)	IJV (1) Fac. (1) EJV (3)
Hanasono et al.^19^	ALT (42) RFF (32) Fibula (26) RA (9) LD (7) Serratus anterior muscle (5) Iliac crest (2) Jejunum (1) Ulnar forearm (1) Lateral arm (1)	ALT (66) RFF (23) Fibula (34) RA (11) LD (5) Serratus anterior muscle (3) Iliac crest (1) Jejunum (3) Ulnar forearm (1) Scapula (2) Internal mammary perforator (1) Gracilis muscle (1) Groin (1)		N/A/ 98.7%	STA (11) TCA (4) Fac. (71) ECA (13) Lin. (15) STempA (12)	STA (25) TCA (15) Fac. (42) ECA (22) Lin. (4) STempA (16) Thoracoacromial (1) Internal mammary (5)	STA (5) TCA (4) Fac. (5) ECA (5) Lin. (2) STempA (1)	IJV (47) Fac. (56) EJV (11) TCV (5) STempV (11)	IJV (43) Fac. (44) EJV (20) TCV (7) STempV (13) Thoracoacromial (1) Cephalic (1)	IJV (6) Fac. (7) EJV (6) TCV (3) STempV (1)
Ross et al.^20^*	N/A	ALT (22) RFF (34) Fibular (21) RA (15) Scapular (10) LD (12) Deep inferior epigastric perforator (3) Lateral arm (3) DCIA(1) Groin (1) Jejunal (1)	N/A	N/A/ 90%	N/A			N/A		
Alam et al.^21^*	RFF (13) Fibular (16) RA (2) Iliac crest (1) Jejunum (1)	RFF (11) Fibular (20) RA (2) LD (1)	N/A	N/A/ 100%	N/A	STA (4) TCA (3) Fac. (20) ECA (3) Lin. (2) Thoracoacromial (1)	N/A	N/A	IJV (9) Fac. (14) EJV (6) TCV (4)	N/A
Mccarn et al.^22^	RFF (41) Fibular (12) RA (3) ALT (3) Ulnar (4) Jejunum (1) LD (1) Unknown (1)	RFF (37) Fibular (15) ALT (6) Ulnar (1) Jejunum (3) LD (2) Scapular (1) Lateral arm (1)	N/A	N/A/97%	N/A	cSTA (8) ECA (11) Radial (28) Thyrocervical trunk (5) STempA (8) Internal mammary (1)	N/A	N/A		
Varvares et al.^23^	RFF (16) Fibular (5) Lilac crest (9) RA (5) LD (1)	RFF (15) Fibular (13) Lilac crest (2) RA (5) LD (1)	RFF (1) Fibular (1) Lilac crest (1) Rectus (1)	N/A/ 94%	N/A			N/A		
Knoetgen et al.^24^	Fibular (3) RA (3) RFF (2) ALT (2) Scapular (1) Parascapular (1)	Fibular (3) RA (4) RFF (2) ALT (3) LD (1)	N/A	100%/ 85%	Fac. (9) STA (1) STempA (1) Lin. (1)	Fac. (7) STA (1) STempA (2) Lin. (1) forearm pedicle (1) pharyngeal (1)	N/A	Fac. (10) STV (1) IJV (1)	Fac. (8) IJV (2) EJV (1) STempV (2)	N/A
Nakayama et al.^27^	Omentum (3) RA (2) RFF (2) Composite (1)	RA (3) ALT (2) RFF (1) Fibular (1) LD(1)	N/A	N/A/100%	Gastroepiploic (3) Radial (3) Inferior epigastric (2)	Gastroepiploic (3) Radial (3) Inferior epigastric (2)	N/A	Gastroepiploic (3) Radial (3) Inferior epigastric (2)	Gastroepiploic (3) Inferior epigastric (2) EJV (2) Cephalic (1)	N/A
Demirkan et al.^25^	RFF (5) Composite (1)	Fibular (2) RFF (3) Rectus femoris (1)	ALT (2) RFF (1) RA (1) Composite (2)	100%/ 100%	STA (3) Lin. (2) Fac. (1) STempA (1)	STA (3) Fac. (2) STempA (1) CTA (1)	STA (3) Fac. (1) STempA (1) CTA (2)	IJV (4) Lin. (1) STV (1) STempV (1)	IJV (4) Fac. (2) STempV (1)	IJV (3) EJV (2) Fac. (1) STempV (1)
Demirkan et al.^28^	RFF (22) Fibular (5) RA (2) Composite (6)	RFF (15) Fibular (10) RA (4) ALT (1) Jejunum (1) Composite (3) Tensor fasciae latae (1)	N/A	97.3%/ 94.6%	N/A			N/A		
Pickford et al.^26^	RFF (5) Dorsalis pedis (1)	RFF (6)	N/A	83.7%/100%	STA (2) Lin. (1) Fac. (3)	STA (1) Lin. (1) Fac. (3) EJA (1)	N/A	EJV (2) STempV (1) STV (1) Fac. (1) Unnamed (1)	IJV + Ant.JV (2) STV + Ant.JV (1) IJV (1) Fac. (1) Fac.+retro mandibular (1)	N/A

ALT, Anterolateral Thigh; RFF, Radial Forearm Flap; DCIA, Deep Circumflex Iliac Artery; PTF, Posteromedial Thigh Flap; LD, Latissimus Dorsi; RA, Rectus Abdominis; MSAP, Medial Sural Artery Perforator; DIEP, Deep Inferior Epigastric Perforator; STA, Superior Thyroid Artery; STempA, Superficial Temporal Artery; ECA, External Carotid Artery; TCA, Transverse Cervical Artery; APA, Ascending Pharyngeal Artery; Lin., Lingual; Fac., Facial; STV, Superior Thyroid Vein; STempV, Superficial Temporal Vein; IJV, Internal Jugular Vein (include the branch); EVJ, External Jugular Vein; TCV, Transverse Cervical Vein; Ant.JV, Anterior Jugular Vein.

a In this article, just free flaps included in sequential flaps to analyses.

b At the first time, 42 free tissue transfers were performed to reconstruct the defects because of two cases with large defects.

c 61 patients in this study had sufficient data to determine with certainty which arteries were used in the anastomosis.

Only 2 articles reported the duration of operation. Moratin et al.^12^ reported the first and second mean operative time was 423 and 427 min respectively. But Demirkan et al.^28^ reported 351 and 562 min. 3 articles^12^^,^^25^^,^^28^ reported the mean length of hospitalization for each time. For the initial procedure, the mean hospitalization durations were 22.4 days, 20 days, and 24.1 days, respectively. Hospitalization durations were generally higher for subsequent procedures compared to the initial procedure. 16 articles^12^^,^^14^, ^15^, ^16^, ^17^, ^18^, ^19^, ^20^, ^21^, ^22^, ^23^, ^24^, ^25^, ^26^, ^27^, ^28^ reported flap-related postoperative complications, with a total of 313 cases, of which the most common were revision for various reasons (19.2%), total necrosis (17.3%), and arteriovenous thrombosis (10.9%), followed by wound infection (9.9%) and delayed wound healing (7.7%). 10 articles,^13^^,^^14^^,^^16^^,^^18^^,^^19^^,^^21^, ^22^, ^23^, ^24^ 28 reported the follow-up results, involving 443 patients. The mean follow-up time was 40.8 months. And among them, 259 (58.5%) survived and 19 (4.3%) lost to follow-up by the respective follow-up dates. Detailed perioperative outcomes and follow-up results are shown in Table 5.

**Table 5:** Outcomes reported by articles.

Articles	Survive outcomes			Perioperative outcomes		
	Mean follow-up (mon)	5-year survival rate	Alive at follow-up (nº)	Operation time (first/second) (min)	Length of hospitalization (d)	Complication (nº)
Moratin et al.^12^	N/A			423/427	Second (22.4 ± 14.3)	Anastomosis Revision (44); Flap loss (24); Wound healing disorders (13); Bleeding (7); Fistula (6); Hematoma (3); Death (4)
					Third (23.7 ± 18.5)	
					≥ Fourth (27.9 ± 21.9)	
Lai et al.^13^	25.6	N/A	26	N/A		Wound healing poor (6); Flap failure (5); Vessel thrombosis (5); Wound infection (3); Partial flap necrosis (3); Fistula (2); Bleeding (2)
Jung et al.^14^	88.3^a^	62.5	5	N/A		Wound dehiscence (6); Bleeding (1)
Vamadeva et al.^15^	N/A			N/A		Partial flap necrosis (3); Infection (2); Others (4)
Chung et al.^16^	44.2	50%	29	N/A		Revision (10); Partial flap necrosis (2); Hematoma (1)
Chiu et al.^17^	N/A			N/A		Thrombosis (8); Infection (5); Delayed wound healing (4); Total necrosis (4); Partial necrosis (1)
Mochizuki et al.^18^	84.5	60.2%	19 (1 lost)	N/A		Infection (6); Thrombosis (1); Fistula (1)
Hanasono et al.^19^	47.5	75.4	87	N/A		Wound dehiscence (15); Infection (13); Hematoma (7); Thrombosis (7); Fistula (6); Seroma (4); Total necrosis (2); Partial necrosis (2); Skin Graft loss (2)
Ross et al.^20^	N/A					Failure (12); Thrombosis (9); Partial necrosis (5); Death (3); Fistula (3)
Alam et al.^21^	13	N/A	21	N/A		Hematoma (1)
Mccarn et al.^22^	14.7	N/A	28 (17 loss)	N/A	9	Hematoma (6); Revision (5); Total necrosis (2); Partial necrosis (1); Bleeding (1)
Varvares et al.^23^	45.5	N/A	20	N/A		Total necrosis (2)
Knoetgen et al.^24^	23.4	64.2	5 (1 loss)	N/A		Wound healing delay (1); Flap failure (1); Others (1)
Nakayama et al.^27^	N/A			N/A		
Demirkan et al.^25^	N/A			N/A	First 20	Fistula (1); Partial necrosis (1); Revision (1)
					Second (41)	
					Third (47)	
Demirkan et al.^28^	37.5	N/A	19	351/562	First 24.1	Total necrosis (1); Hematoma (3); Infection (2); Partial necrosis (1); Bleeding (1); Fistula (1); Thrombosis (1)
					Second 40	
Pickford et al.^26^	N/A			N/A		Thrombosis (2); Poor filling of vein (1); Failure (1)

In addition, 2 articles reported the relationship between preoperative radiotherapy and surgical outcomes. Demirkan et al.^25^ did not consider a history of radiotherapy as a contraindication for vascular exploration of the ipsilateral recipient area. All 6 cases they reported had undergone radiotherapy after the previous flap surgery, and the sequential flaps surgeries were all successful. 9 of the 26 cases reported by Mochizuki et al.^18^ underwent preoperative radiotherapy, which was considered to induce mesenchymal and vascular microscopy to avoid separation difficulties, and their vascular anastomoses were performed on the nonirradiated side; 4 of the 7 cases who developed postoperative complications received preoperative radiotherapy, and there was no significant difference between preoperative radiotherapy and the development of postoperative complications. However, the authors suggested that cases with a history of preoperative radiotherapy may have a high risk of postoperative wound infection.

And only 1 article discussed the management of the previous flap during the re-flap surgery. Hanasono et al.^19^ reported that 30 subsequent flaps reusing the recipient vessels of the previous flap were successful. Among these 30 cases, the previous flap was completely resected in 20 cases, partially resected in 4 cases, and preserved in 6 cases. All unresected or partially resected flaps were survived after dissection of the vascular pedicle.

## Discussion

Free flaps have become a widely used reconstructive option for repairing large tissue defects caused by tumor resection in HNC patients, with a high overall success rate, reported in some studies to exceed 95%, and contributing to the restoration of the tissue morphology, associated function and improved postoperative quality of life.^29^^,^^30^ Despite the high success rate of free flaps, in some patients, always have repaired tissue reappearing with large defects for various reasons, and the most common of which in our review is recurrence of the primary tumor. In these patients, the decision between undergoing a second or additional free flap reconstruction surgery versus opting for conservative treatment (such as radiotherapy or palliative care) is particularly important.

This review demonstrates that the success rate of sequential flaps can reach 94.6%, which is comparable to the success rate of single flap reconstruction of the head and neck in previous studies. The majority of patients included in our review underwent second or multiple reconstructions due to cancer recurrence, suggesting that sequential flaps are a feasible option for HNC patients requiring reconstruction again. In studies that have reported the interval time between two reconstructions, the span of interval time greatly, ranging from as short as 2-days^12^ to as long as 12-years.^22^ There is no definitive restriction on the timing for sequential flap reconstructions. Only 1 article^12^ has compared the impact of different interval time for sequential flap reconstruction on the flap success rate. The results showed that the success rate of sequential flap reconstruction within 30 days after the initial surgery was slightly higher than that after 30 days, but the difference was not statistically significant. However, flap failure still occurs, and the reported reasons for such failures include: reoperation for cancer recurrence,^12^ patients with multiple surgeries are more likely to fail as the number of surgeries increases^13^ and radiotherapy administered between surgeries.^14^ Notably, a history of radiotherapy has been suggested by several investigators as a limiting factor for flaps success, but reliable statistical evidence is still lacking. For patients undergoing a second or multiple head and neck reconstruction with flaps, the presence of scar tissues and changes in anatomical structure due to the previous surgery, lack of volume of the soft tissues, lack of adequate bone support, and the scarcity of blood vessels available for anastomosis or the quality of the blood vessels because of the radiotherapy may all be contributing to the factors of the flap failure.

The majority of patients need reconstruction again are cancer recurrence patients, we cannot change the fact that these patients have already undergone surgery or radiotherapy, therefore, the choice of appropriate flaps and vessels for anastomosis plays a crucial role in determining the success of flap. However, there is no definitive answer regarding the best flap or vessel choice, as each patient’s condition is unique. Some suggestions from the articles include: the radial forearm flap may be preferred for intraoral repair, the fibula-associated flap may be suitable for larger mandibular defects and the rectus abdominis or anterolateral femoral flap for larger soft tissue defects^24^; if the vessel pedicles from the previous anastomosis are long enough for the donor area and blood flow well, they can be used again,^17^^,^^19^ if not, the transverse cervical vessels or the contralateral vessels can be considered, and if the contralateral vessels are used, the arteries can be considered to be the superior thyroid artery and facial artery, and the veins can be considered to be the facial vein and the internal jugular vein, and the larger vessel caliber and longer vessel pedicles are associated with higher success rates;^13^^,^^15^^,^^17^, ^18^, ^19^^,^^22^^,^^23^^,^^25^ flap success may be reduced if vein grafts are used.^20^ Opinions are divided on the necessity of preoperative assessment using preoperative angiography and other related examinations. Some authors advocate that it is necessary^21^^,^^27^ and others do not.^23^

The most common postoperative complication is revision due to a variety of reasons, including thrombosis, hematoma, hemorrhage, etc., as well as total or partial necrosis, delayed wound healing, bleeding, wound infection, etc. It is important that enhanced perioperative management and timely management for early symptoms (like flap color turning to pale, increasing drainage and secretion from wounds). Only one article mentioned risk factors for complications: patients with diabetes and a history of smoking were more possibly to have slow wound healing and transient cardiac or respiratory insufficiency, and a higher incidence of complications in composite flaps than in patients reconstructed with a single flap, but the number of patients with composite flaps remains small and still require further investigation. Similarly, there was limited reporting of postoperative follow-up outcomes and only 2 articles discussed the related functional recovery after surgery. 1 article reported that among patients who underwent oropharyngeal reconstruction, 90.1% had articulation clarity of >80% and 81.6% were able to eat independently,^19^ and the other article reported that among surviving patients who underwent oropharyngeal reconstruction, 20.7% could eat solid meals, 24.1% could eat only soft meals and 12.1% were unable to eat anything; in terms of speech, 10% had speech impairment, and 24.1% were unable to speak.^16^

In addition, none of articles included in this review reported whether to assess the patients’ postoperative psychological changes and quality of life. For patients who have had surgeries, especially those with recurrent cancers, it is important that whether the reoperation actually benefits their outcomes or prognosis. This review also has some limitations: The articles included were all retrospective studies, the majority of articles had a moderate risk of bias according to the MINORS criteria. Due to the low evidence level of most studies and the presence of significant heterogeneity, as well as incomplete key indicators provided in some studies (such as missing standard deviations, confidence intervals, etc.), a meta-analysis was not conducted in this review. In the future, if more homogeneous studies become available, they will helpful for a meta-analysis. Few articles reported the risk factors related to prognosis or outcomes (except postoperative complications);and the included studies were published over a long period (1995–2021), during which surgical techniques and perioperative management may have advanced significantly; and there may be a large variation in operative time and length of hospital stay, which may not have been well assessed in our study for related outcomes; finally, no article reported whether alternative options were available to patients, such as palliative care, and it was not possible to assess whether patients had better or worse outcomes compared with the alternatives.

## Conclusion

Sequential flaps are an optional and effective reconstructive modality for HNC patients. However, prospective studies are needed to explore prognostic-related risk factors and better determine the suitability of sequential flaps for individual patients. Additionally, since reconstruction impacts both aesthetics and function, it is crucial to emphasize during preoperative discussions that postoperative outcomes may not fully align with patient expectations.

## ORCID ID

Zepeng Xu: 0009-0009-5046-5862

Mailudan Ainiwaer: 0009-0004-7386-8457

Zheng Jiang: 0000-0001-7160-4512

Fei Chen: 0000-0003-2152-368X

## Funding

The 1.3.5 Project for Disciplines of Excellence, West China Hospital, Sichuan University (Award Number: 20HXJ5014).

## Declaration of competing interest

The authors declare no conflicts of interest.
